# Soluble Glycoprotein 130Fc Reduces Controlled Cortical Impact-Induced Cognitive Deficits in Rats

**DOI:** 10.1177/2689288X251377032

**Published:** 2025-09-17

**Authors:** Ashley L. Russell, Emma G. Dimeo, Anisha Mandava, Lara Nasser, Natalie Convertino, Sneha Padamati, Ian G. Gober, Julie A. Scott, Vritika S. Patel, Jenna C. Carlson, Patrick M. Kochanek, Amy K. Wagner

**Affiliations:** ^1^ Department of Physical Medicine and Rehabilitation, School of Medicine, University of Pittsburgh, Pittsburgh, Pennsylvania, USA.; ^2^ Safar Center for Resuscitation Research, University of Pittsburgh, Pittsburgh, Pennsylvania, USA.; ^3^ Department of Neuroscience, School of Arts and Sciences, University of Pittsburgh, Pittsburgh, Pennsylvania, USA.; ^4^ Department of Biostatistics and Health Data Science, School of Public Health, University of Pittsburgh, Pittsburgh, Pennsylvania, USA.; ^5^ Department of Human Genetics, School of Public Health, University of Pittsburgh, Pittsburgh, Pennsylvania, USA.; ^6^ Department of Critical Care Medicine, School of Medicine, University of Pittsburgh, Pittsburgh, Pennsylvania, USA.; ^7^ Clinical and Translational Science Institute, University of Pittsburgh, Pittsburgh, Pennsylvania, USA.

**Keywords:** behavior, inflammation, interleukin-6, Morris water maze, soluble glycoprotein 130, traumatic brain injury

## Abstract

Traumatic brain injury (TBI) can lead to cognitive dysfunction, with underlying mechanisms poorly understood. Interleukin (IL)-6, particularly via soluble IL-6 receptor (sIL-6R) trans-signaling, can exacerbate neurodegeneration. Soluble glycoprotein (sgp)130 inhibits this pathway, reducing neuroinflammation and improving cognition in a mouse TBI model. In this study, we evaluated sgp130Fc on cognitive recovery and neural damage using a severe controlled cortical impact (CCI) injury model in rats. Male rats (*N* = 37) underwent CCI (6-mm flat-tip impactor; 4.0 m/sec; 2.8 mm deformation) or sham procedures and received sgp130Fc (10 mg/kg) or vehicle (VEH) intraperitoneally every 3 days starting 1 day post-injury (dpi). Motor function (beam walk and balance; 1–7 dpi), and spatial learning and memory (Morris water maze [MWM], 14–19 dpi) were assessed using a repeated measures analysis of variance followed by a Tukey’s post hoc analysis. Lesion volume was assessed (21 dpi) using a two-tailed *t*-test. CCI rats exhibited transient motor deficits not influenced by sgp130Fc. CCI + VEH rats had longer latencies and path lengths to the submerged platform versus shams (*p* < 0.05). On 18 dpi, CCI + sgp130Fc rats had shorter latencies (*p* = 0.04) and path lengths (*p* = 0.02) versus CCI + VEH rats. CCI + VEH rats displayed more inefficient swim strategies than CCI + sgp130Fc rats. CCI + VEH, but not CCI + sgp130Fc rats, had significantly worse performance on the MWM visible platform trials, particularly with respect to path length, suggesting, along with the swim strategy data, possible benefit with spatial navigation when the platform is utilized as a proximal cue. sgp130Fc did not significantly reduce lesion volume. These findings support sgp130Fc as a potential therapeutic for cognitive recovery following TBI.

**Figure:**
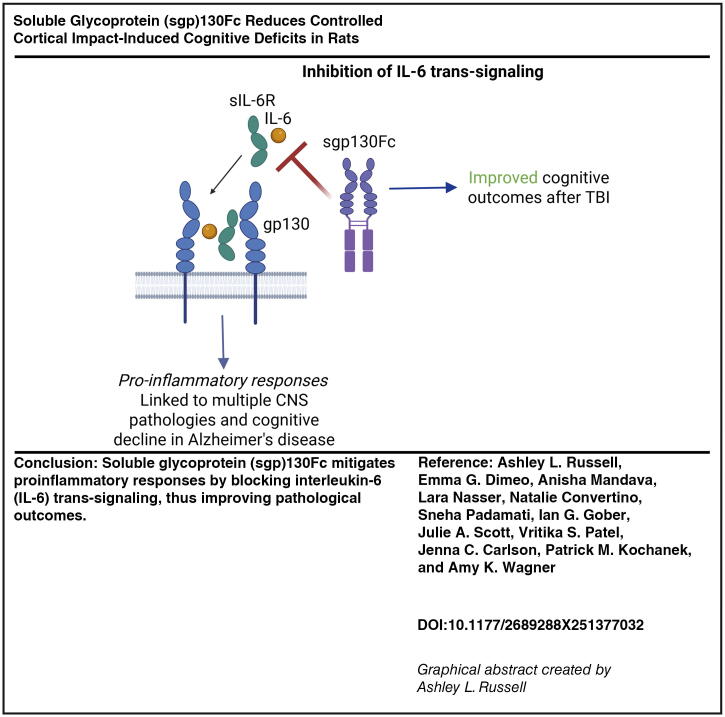


## Introduction

Traumatic brain injury (TBI) survivors often face cognitive and motor impairments.^1^ These deficits vary in severity and chronicity, impacting overall quality of life.^1^ A key contributor to these impairments is chronic uncontrolled inflammation,^2,3^ a secondary injury response,^4,5^ and for which interleukin (IL)-6 pathology has been implicated clinically in the acute^6–10^ and chronic^2^ phases post-injury. Despite progress in the TBI field in understanding acute, subacute, or chronic phases of patient management and diagnosis,^11,12^ there are no effective disease-modifying therapies for long-term TBI outcomes.

Modulating early and prolonged inflammatory responses after TBI may improve long-term outcomes.^4^ One key target is IL-6, an immunomodulatory cytokine elevated after TBI and linked to poor outcomes.^2,6–10,13–16^ Clinically, IL-6 is elevated in the days after TBI and is negatively associated with recovery outcomes.^9,17^ Higher cerebrospinal fluid IL-6 over the first 5 days post-injury (dpi) is associated with increased serum IL-6 levels and increased odds of an unfavorable Glasgow Outcome Scale score at 6 months post-injury.^8^ A higher serum inflammatory burden after severe TBI, as measured by an increased IL-6/IL-10 ratio (pro-inflammatory/anti-inflammatory), is associated with an increased odds of unfavorable outcome at 6 months post-injury.^2^ Preclinical literature also demonstrates acute increases in systemic and central nervous system (CNS) IL-6 after TBI across various injury models and severities.^18–22^ Thus, the potential role IL-6 plays with both systemic and CNS dysregulation and pathology after TBI makes it a possible therapeutic target to promote neurorecovery.

Among healthy individuals, IL-6 levels are low; however, levels can rise dramatically—up to six orders of magnitude—in disease, highlighting its role in disease pathology.^23^ IL-6 is implicated in immune system pathologies including rheumatoid arthritis, cytokine release syndrome, Crohn’s disease, irritable bowel syndrome, cancer, sepsis, and COVID-19.^24–27^ IL-6 is also linked to neurodegenerative diseases such as Alzheimer’s disease (AD), multiple sclerosis, and Parkinson’s disease. In AD, higher IL-6 correlates with poorer cognition and hypothalamic/hippocampal volumes.^28^ In a mouse AD model, IL-6 neutralization improved memory and normalized IL-6 plasma levels.^28^

However, IL-6 affects both homeostasis and disease, making targeting IL-6 a nuanced process. Critical to this point, the involvement of IL-6 in both anti-inflammatory and pro-inflammatory processes^29^ is dependent on the signaling pathway. IL-6 can act via classical or trans-signaling. In classical signaling, IL-6 binds to membrane-bound IL-6 receptor (mIL-6R) and membrane-bound glycoprotein 130 (gp130). In trans-signaling, soluble IL-6R (sIL-6R), which is generated by constitutive and (e.g., injury) inducible proteolytic cleavage by ADAM10 and ADAM17, respectively,^30,31^ forms a complex with IL-6 to activate gp130.^32^ gp130 is ubiquitously expressed, but IL-6R is limited to specific cell types like hepatocytes, T cells, and leukocytes.^23^ Thus, trans-signaling can occur in virtually all cell types, while classical signaling is limited to IL-6R-expressing cells. Classical signaling is associated with anti-inflammatory and protective processes including regeneration, bacterial infection defense, and cell proliferation.^23^ Trans-signaling induces pro-inflammatory responses and is associated with multiple CNS pathologies.^23,25,33^

IL-6 signaling can be blocked through antibodies to the receptor complex or inhibitors of signaling molecules, but these may have off-target effects. For example, inhibition of Janus kinase/signal transducer and activator of transcription (JAK/STAT)—downstream signaling molecules—can impact many signaling cascades beyond IL-6. JAK inhibitors such as baricitinib, tofacitinib, and filgotinib are currently in clinical trials for autoimmune, allergic, and inflammatory diseases.^34^ Tocilizumab and sarilumab, IL-6R antibodies to IL-6R,^35–37^ block both IL-6 signaling pathways, which may limit efficacy or cause unintended risks such as blocking protective classical signaling and causing immunosuppression that leads to detrimental clinical outcomes such as increased nosocomial infection risk. A promising alternative is soluble (s)gp130Fc, a fusion of soluble glycoprotein (sgp)130 and the Fc portion of immunoglobulin (IgG). It binds the IL-6/sIL-6R complex and impedes gp130 signaling, thus selectively blocking IL-6 trans-signaling without affecting classical signaling.^32,38,39^ Inhibition of IL-6 trans-signaling benefits several disease states including acute pancreatitis,^40^ bone fracture healing,^41^ myocardial infarction,^42^ and TBI.^43^ We previously showed in a mouse TBI model that sgp130Fc reduced TBI-induced cognitive deficits, anxiety, and brain inflammation.^43^ To begin to extend this work, we hypothesized that targeting IL-6 trans-signaling may reduce TBI-associated behavioral deficits in a severe controlled cortical impact (CCI) model in rats.

## Methods

### Animals

Adult (*N* = 37; 290–325 g) male Sprague–Dawley rats (Envigo) were pair-housed with *ad libitum* access to food and water and maintained in a temperature-, humidity-, and light-controlled environment. Following 1 week of acclimatization, rats were randomly assigned to one of four experimental groups: (1) Sham + vehicle (VEH; *n* = 9), (2) Sham + sgp130Fc (*n* = 9), (3) CCI + VEH (*n* = 9), and (4) CCI + sgp130Fc (*n* = 10). Rats were randomly assigned to a treatment group after they were pretrained on motor tasks but prior to any experimental intervention.

### Controlled cortical impact model

CCI was performed under aseptic conditions.^44–47^ Rats were anesthetized with isoflurane (4% induction, 2% maintenance in N_2_O:O_2_), intubated, secured in a stereotaxic frame, and mechanically ventilated. Core body temperature was maintained at 37 ± 0.5°C. Betadine was applied to the surgical site. A 6-mm craniotomy was made over the right hemisphere with a dental drill and subsequently enlarged to approximately 10 mm in diameter. The CCI was performed using a 6-mm flat impactor tip (2.8 mm tissue deformation from dura; 4 m/sec; 100 m/sec dwell time; 20° offset) such that the impactor was perpendicular with the dural plane. Immediately after impact, anesthesia was discontinued. The craniotomy was left open and the incision sutured. Rats were extubated and assessed for acute neurological outcome using righting reflex. Sham rats underwent all surgical procedures, except the impact injury.

### sgp130Fc administration

Rats received intraperitoneal (i.p.) injections of recombinant rat gp130Fc chimera protein (sgp130Fc; R&D Systems; 10 µg/kg) in sterile phosphate-buffered saline (PBS) starting 1 dpi and administered every 3 days. The dose and schedule were based on previous data^43^ and the 72-h half-life of the sgp130Fc.^47^ VEH-treated rats received PBS injections following the same schedule. Injections were administered after behavioral testing on test days.

### Motor function

**FIG. 1.**
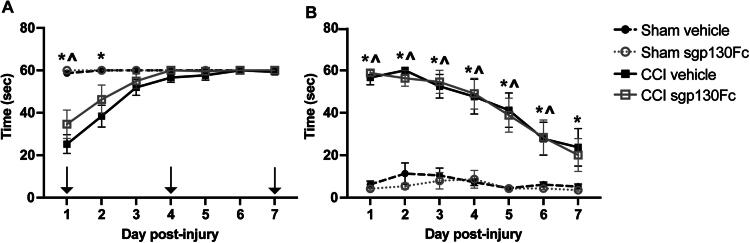
Motor function 1–7 dpi. **(A)** Time spent balancing on the beam. **(B)** Time to traverse the beam walk. Arrows depict sgp130Fc treatment on 1, 4, and 7 dpi (*after testing*). Points represent mean ± SEM. *p < 0.05 Sham + VEH versus CCI + VEH; ^*p* < 0.05 Sham + sgp130Fc versus CCI + sgp130Fc. SEM, standard error of the mean; VEH, vehicle; CCI, controlled cortical impact.

Motor function was assessed on the beam balance and beam walk 1–7 dpi (Fig. 1).^48–50^ Rats were pretrained and baseline tested before surgery. Beam balance performance was measured as the time (max 60 sec/trial) each rat remained on an elevated (90 cm) beam (1.5 cm wide; 34 cm long). Beam walk was assessed by time (max 60 sec/trial) and distance to cross a beam (2.5 cm wide; 100 cm long). A bright light and noise were used as a stimulus to promote beam traversal and entrance into a goal box at the opposite end. Each task included three trials/day.

### Spatial learning and memory

**FIG. 2.**
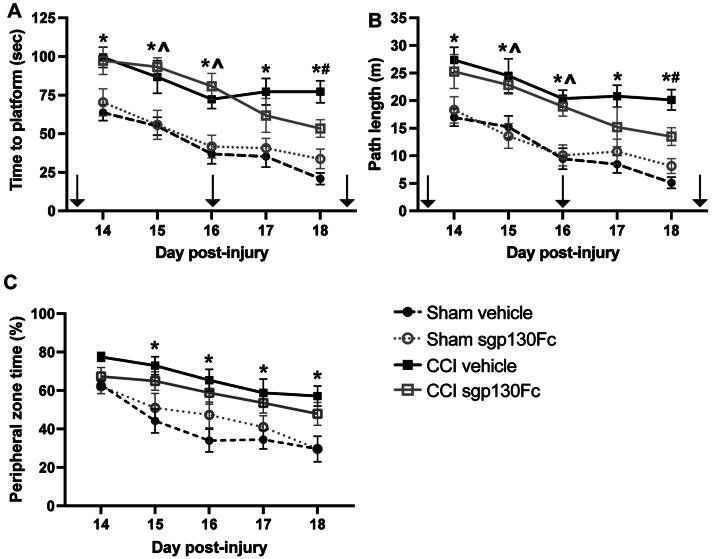
MWM spatial learning and memory during acquisition trials (14–18 dpi). **(A)** Latency to locate the submerged platform. **(B)** Path length traveled to locate the submerged platform. **(C)** Time spent in the peripheral (thigmotaxis) zone. Points represent mean ± SEM. *p < 0.05 Sham + VEH versus CCI + VEH; ^*p* < 0.05 Sham + sgp130Fc versus CCI + sgp130Fc; #*p* < 0.05 CCI + VEH versus CCI + sgp130Fc. MWM, Morris water maze; SEM, standard error of the mean; VEH, vehicle; CCI, controlled cortical impact.

Spatial learning was assessed using the Morris water maze (MWM) on 14–18 dpi (Fig. 2).^46,50–53^ The maze consisted of a pool (180 cm diameter; 60 cm high) and a Plexiglass platform (10 cm diameter; 26 cm high). The pool was filled with water (26 ± 1°C) to a depth of 28 cm such that the hidden platform was submerged 2 cm below the water surface. Extra-maze cues were present. Each rat was tested on four trials/day with 4-min intertrial intervals for 5 consecutive days (14–18 dpi; 120 sec/trial max). For each trial, the rat started from a different pseudo-randomized cardinal position of the pool.

**FIG. 3.**
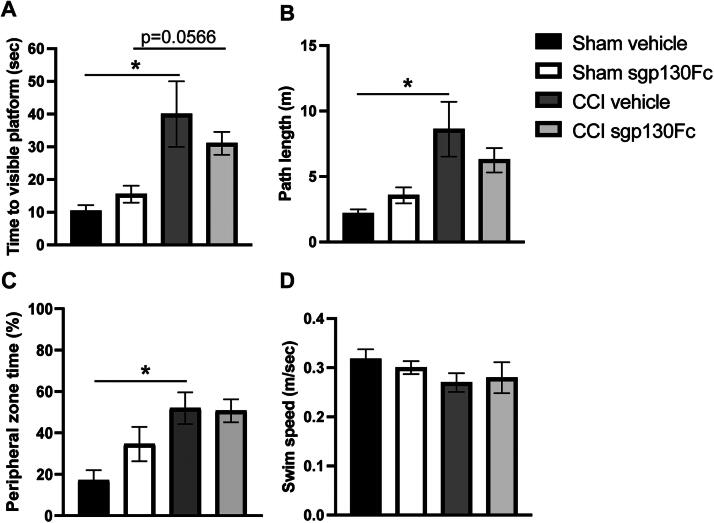
MWM visible platform trials (19 dpi). **(A)** Latency to locate the visible platform. **(B)** Path length traveled to locate the visible platform. **(C)** Time spent in the peripheral (thigmotaxis) zone. **(D)** Average swim speed over all testing trials (14–19 dpi). Bars represent mean ± SEM. *p < 0.05. MWM, Morris water maze; SEM, standard error of the mean.

Visible platform (VP) trials were completed 19 dpi (Fig. 3).^46,51–53^ The platform was raised 2 cm above the surface of the water for visibility. Each rat was tested on four trials with 4-min intertrial intervals (120 sec/trial max). The latency to find the platform, peripheral (thigmotaxic) zone time, path length, and swim speed were recorded and analyzed using ANY-maze software.

**FIG. 4.**
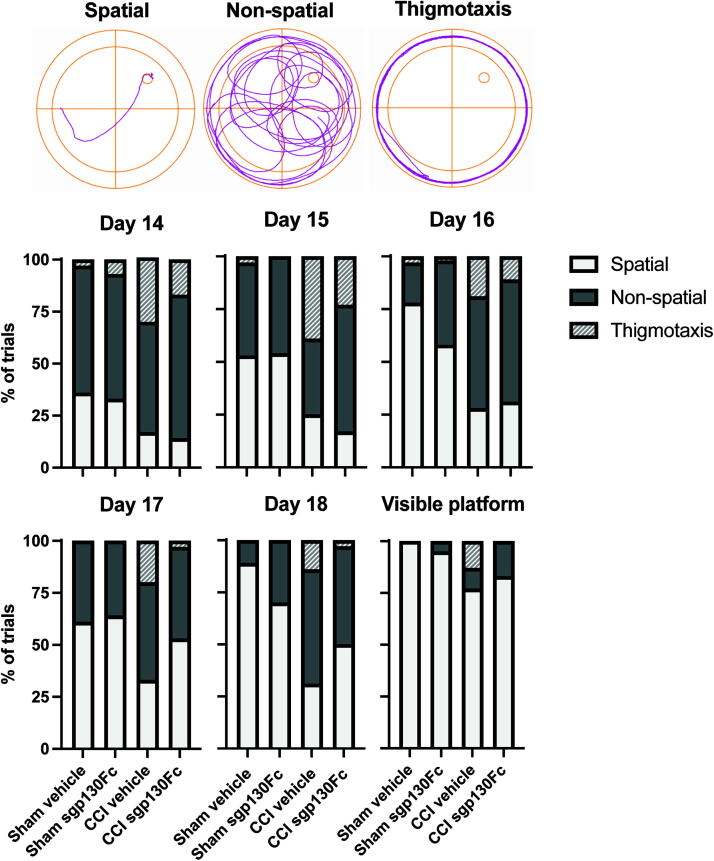
MWM swim strategy during acquisition (14–18 dpi) and VP (19 dpi) trials. Each trial was scored as either (1) spatial, (2) nonspatial, or (3) thigmotaxis, and the percent of trials is represented for each experimental group. MWM, Morris water maze; VP, visible platform.

Search strategies across all MWM trials (*n* = 874) were qualitatively analyzed using ANY-maze tracking plots. Two blinded experimenters classified the predominant swim strategy per trial.^46,52,54,55^ Discrepancies were resolved by a third experimenter. Classifications were based on swim path complexity and platform-finding efficacy and grouped as spatial, nonspatial, or thigmotaxis (Fig. 4). Spatial strategies included direct and self-orienting paths. Nonspatial included scanning, circling, or random searches. Thigmotaxis was defined as wall-hugging behavior (Fig. 4). Strategy use per rat was calculated as a percentage of total trials.

### Tissue preparation

On 21 dpi, rats were deeply anesthetized with 4% isoflurane in 2:1 N_2_O:O_2_ and transcardially perfused with 200 mL of PBS (pH 7.4) followed by 300 mL of 4% paraformaldehyde (PFA). Brains were extracted and postfixed in 4% PFA, dehydrated with alcohols, and embedded in paraffin. Coronal sections (5-mm thick) were cut at 0.5-mm intervals using a microtome throughout the lesion and mounted onto microscope slides.

### Cortical lesion volume

Sections were deparaffinized, rehydrated, and stained with cresyl violet for cortical lesion volume analysis. Cortical lesion volumes (mm^3^) were quantified by two observers blinded to experimental conditions using image analysis software (MCID; Imaging Research). Three sections per 0.5-mm interval throughout the lesion were analyzed and averaged. Lesion volume and tissue volume loss were expressed as a percent of the contralateral (“non-injured”) hemisphere.^56,57^

### Statistical analysis

Data were analyzed in GraphPad Prism 10 and reported as mean ± standard error of the mean (SEM). Distribution and variance were assessed. Beam balance, walk, and MWM acquisition were analyzed using repeated-measures analysis of variance (ANOVA) for injury, time, and injury × time interaction effects, followed by Tukey’s post hoc test. One-way ANOVA with Tukey’s test assessed intraday injury effects (acquisition) and VP outcomes. Swim strategies (14–18 dpi) were analyzed per animal. The relationship between the group and thigmotaxis trial count was tested with the Kruskal–Wallis test, followed by Sidak post hoc. Due to its rarity, thigmotaxis occurrence (yes/no) was tested using a Fisher’s exact test. Righting reflex time and lesion volume were analyzed using equal-variance two-tailed *t*-tests. Significance was set at 0.05, with post hoc comparisons maintaining a family-wise error rate of 0.05.

## Results

### There was no significant difference in righting reflex between CCI cohorts

Average righting reflex time was similar between CCI + VEH (367 ± 31.20 sec) and CCI + sgp130Fc (426 ± 36.02 sec) rats and was not statistically different between groups (*p* = 0.2324).

### CCI-induced motor deficits resolved by 7 dpi

There was a significant interaction between injury and time post-injury (*p* < 0.0001) for beam balance and beam walk (Fig. 1). CCI rats had a shorter balance duration versus shams at 1 and 2 dpi, with these deficits resolving by 3 dpi (Fig. 1A). In the beam walk, CCI rats took longer to traverse the beam than shams, nearing recovery by 7 dpi (Fig. 1B). Only CCI + VEH rats took longer versus Sham + VEH rats at 7 dpi (*p* = 0.04). However, CCI + sgp130Fc had no effect on beam walk performance compared with CCI + VEH.

### sgp130Fc improved CCI-induced learning and memory deficits in the MWM

There were significant effects of injury (*p* < 0.0001) and time post-injury (*p* < 0.0005) on MWM latency (Fig. 2A), path length (Fig. 2B), and peripheral zone time (Fig. 2C). CCI + VEH rats had longer latencies than Sham + VEH rats (14–18 dpi; Fig. 2A), indicating CCI-induced learning deficits. CCI + sgp130Fc rats had longer latencies to find the submerged platform than Sham + sgp130Fc rats on 14–16 dpi, but not on 17–18 dpi. By 18 dpi, CCI + sgp130Fc rats had a reduced latency to find the platform versus CCI + VEH rats (*p* = 0.04). A similar pattern emerged in path length (Fig. 2B), demonstrating reduced cognitive deficit with sgp130Fc after injury. CCI rats spent more time in the peripheral zone compared with sham rats. Although not significant, CCI + sgp130Fc rats spent less time in the peripheral zone versus CCI + VEH rats (Fig. 2C).

In the VP, CCI increased the latency, path length, and peripheral zone time (Fig. 3). There was no significant effect of sgp130Fc in CCI or sham rats. There was no effect of CCI or sgp130Fc treatment on average swim speed (Fig. 3D).

Sham rats preferred a spatial strategy in the MWM and were less likely than CCI rats to use a nonspatial strategy (16–18 dpi; Fig. 4). There also was a significant difference in thigmotaxis use across groups (*p* = 0.014). CCI + VEH rats showed the highest likelihood of thigmotaxis (14–16 dpi), followed by CCI + sgp130Fc rats, who were half as likely to use it. Sham rats rarely displayed thigmotaxis. For VP trials, CCI + sgp130Fc rats were significantly less likely to use thigmotaxis compared with CCI + VEH rats. Due to its rarity, we did not have adequate power to appropriately test this outcome as a repeated measure (i.e., strategy employed in each trial within rat).

### Impact of sgp130Fc on lesion volume in CCI rats

**FIG. 5.**
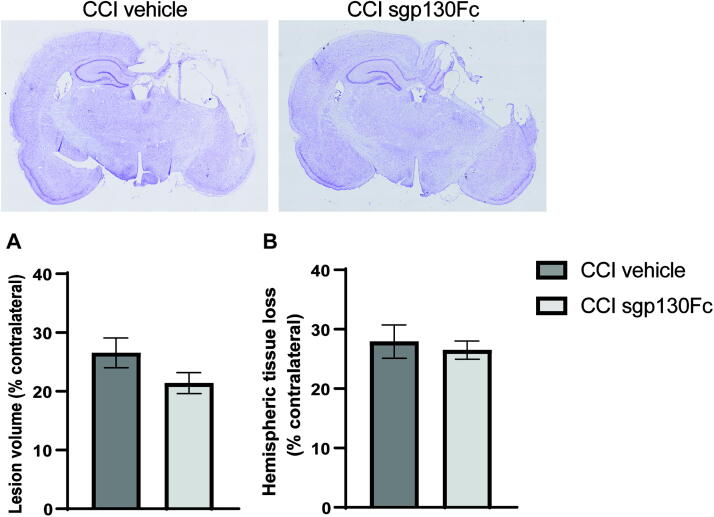
Histopathology after CCI. Top row shows representative images of the lesion site in CCI + VEH (left) and CCI + sgp130Fc (right) rats. **(A)** Lesion volume and **(B)** hemispheric tissue loss at 21 dpi. Bars represent mean ± SEM. CCI, controlled cortical impact; VEH, vehicle; SEM, standard error of the mean.

There was no significant effect of treatment on lesion volume or hemispheric tissue loss comparing CCI + sgp130Fc rats with CCI + VEH at 21 dpi (*p* = 0.1161, Fig. 5A, and *p* = 0.6558, Fig. 5B, respectively).

## Discussion

Uncontrolled inflammation after TBI leads to poor chronic outcomes. Since IL-6 is a reliable stress- and injury-induced cytokine, targeting its signaling mechanism—specifically IL-6 trans-signaling—offers a promising therapeutic opportunity with less side effects (e.g., immunosuppression) than pan-IL-6 inhibition. As expected, CCI induced acute motor deficits (beam walk and beam balance) and impaired cognition (MWM). sgp130Fc improved learning and memory but did not impact motor outcomes or lesion/hemispheric volumes. These findings expand on our previous work,^43^ demonstrating efficacy of sgp130Fc in rats as well as mice.^38^ Overall, our data suggest that intermittent systemic administration of sgp130Fc after TBI may be a viable treatment to promote lasting neurobehavioral recovery.sgp130Fc has shown success in reducing inflammation and affecting outcomes across a variety of conditions. For example, sgp130Fc (olamkicept) is in phase II clinical trials, showing efficacy in treating active ulcerative colitis and inflammatory bowel disease.^32,38^ In the ulcerative colitis trial, 12 weeks of olamkicept improved clinical remission and mucosal healing compared with placebo.^58^ In a mouse sepsis model with elevated IL-6, sgp130Fc enhanced survival, lowered inflammatory cytokines, and reduced cognitive decline.^59^ sgp130Fc also has shown benefits in infectious diseases like COVID-19, reducing symptoms, lung damage, and mortality in mice.^60^ Moreover, we found that intermittent sgp130Fc administration improved neurorecovery after CCI in mice, reducing learning deficits, anxiety-like behaviors, and brain inflammation.^43^ Notably, a recent report suggests neuroprotection in a mouse MCAO model but with sex-specific differences in effective dosing.^61^ Interestingly, the investigators found that females had higher plasma IL-6 and sIL-6R levels at 24 h post-MCAO; thus, a higher dose of sgp130Fc—1 mg/kg in females compared with 0.5 mg/kg in males—was needed to improve functional outcomes at 7 and 28 dpi.^61^ These findings will inform future work exploring sex differences in response to injury and sgp130Fc treatment.

In our rat study, we observed a transient motor deficit after CCI that resolved within a week. sgp130Fc did not reduce motor deficits after CCI. Since IL-6 rises quickly post-injury, earlier sgp130Fc administration should be tested for potential impacts on motor function. Prior work shows that pan-IL-6 inhibition improved motor coordination in the rotarod test 7–12 dpi,^19^ suggesting cognitive-motor tasks like the rotarod may be informative in future studies. However, motor recovery by 7 dpi on beam balance and walking tasks suggests motor deficits did not confound cognitive outcomes in our study, which is further supported by no significant MWM swim speed differences.

At 18 dpi, CCI + sgp130Fc rats showed reduced latency and path length to find the submerged platform versus CCI + VEH rats, suggesting improved cognitive function. This finding reflects a more efficient, spatially directed swim strategy, with less nonspatial and/or thigmotaxis selections. Overall, we show ∼50% reduction in thigmotaxis strategy selection in CCI + sgp130Fc rats versus CCI + VEH rats, with no differences in peripheral zone time. These data suggest that decreased thigmotaxis selection is likely linked to a more spatially directed, efficient swim strategy rather than anxiety-like phenotypes. In a mouse CCI model, intermittent doses of 0.25 mg or 1 mg (i.p.) starting 1 dpi reduced latency and peripheral zone time.^43^ These species differences in sgp130Fc efficacy may be, in part, due to IgG fusion tail immunogenicity as the bioconjugates used differed between rats and mice (mouse IgG_2a_ vs. IgG1, respectively). Additional consideration is needed to understand effector functions^62^ and how this might impact sgp130Fc effectiveness in TBI models. Future studies should also explore sgp130Fc’s effects on additional behavioral outcomes, to address working memory, both short- and long-term retention, cognitive flexibility, and psychological health-related outcomes (e.g., anxiety, anhedonia).sgp130Fc may support cognitive recovery after injury by reducing brain inflammation. We previously showed sgp130Fc lowered chemokines at 21 dpi.^43^ Pan-IL-6 inhibition also reduces inflammation at 1 dpi.^19^ Region- and cell-type-specific effects may contribute. For instance, IL-6-driven STAT3 activation in the hippocampal CA1 impairs memory.^63^ As CA1 was severely damaged in our CCI model, other regions may mediate sgp130Fc’s benefits. Indeed, tocilizumab reduces neuronal death in the hippocampus and cortex post-hemorrhage.^64^ Since microglia express gp130 and are involved in chronic inflammation after TBI,^5,65–67^ they may be key targets with reducing glial-associated IL-6 trans-signaling, wherein cells with constitutive signaling capabilities may be vulnerable to trans-signaling under pathological conditions and in an sgp130:sIL-6R-dependent manner.^68^ Although lesion volume changes were modest, sgp130Fc’s mechanism and neuroanatomical effects in TBI warrant further study in our rat model.

This study has limitations and raises important questions. First, although IL-6 rises sharply after TBI, mIL-6R and gp130 expression are only marginally affected.^32,69^ Due to assay limitations, we could not track biomarkers in our CCI model, though our prior work supports an acute IL-6 rise and the rationale for targeting trans-signaling.^43^ Second, the extent of hippocampal damage in our model limited additional pathology measures. While statistically significant, our findings were modest potentially due to the dose selected and variability in IgG fusion molecule between rat and mouse bioconjugates. Though lower than doses in other models (e.g., sepsis, COVID-19),^59,60,70^ we previously observed cognitive benefits at similar levels in mice.^43^ Dose–response studies are needed to assess pharmacodynamics, pharmacokinetics, and target engagement. Timing may also matter: the blood–brain barrier (BBB) is acutely comprised post-CCI^71,72^ but recovers by 5 dpi,^26^ limiting large molecule access. However, persistent BBB disruption in clinical TBI^73^ suggests this deserves further investigation. Lastly, this study included only males; future work should examine females, given sex differences in neuroinflammatory responses.^5,73,74^

In conclusion, IL-6 trans-signaling is a manipulable target that is associated with multiple inflammatory conditions. We demonstrate that sgp130Fc treatment reduced CCI-induced cognitive deficits in rats, highlighting its therapeutic potential to promote TBI-associated neurorecovery.

## Transparency, Rigor, and Reproducibility

All animals were handled and treated in accordance with the NIH Guide for the Care and Use of Laboratory Animals, and experiments were approved by the University of Pittsburgh IACUC. Sample size calculations were based on previous work.^46,52^ Forty rats underwent procedures; two were excluded for technical reasons and one died. Rats were randomly assigned to groups prior to intervention. Investigators were blinded to experimental group. Data normality was tested using the Shapiro–Wilk test. Study data are available in a FAIR data repository (odc-tbi.org).

## Abbreviations Used

AD: Alzheimer’s disease
ANOVA: analysis of variance
BBB: blood–brain barrier
CCI: controlled cortical impact
CNS: central nervous system
dpi: days post-injury
gp130: membrane-bound glycoprotein130
IL-6: interleukin-6
IgG: immunoglobulin G
i.p.: intraperitoneal
JAK/STAT: Janus kinase/signal transducer and activator of transcription
mIL-6R: membrane-bound interleukin-6 receptor
MWM: Morris water maze
PBS: phosphate-buffered saline
PFA: paraformaldehyde
SEM: standard error of the mean
sIL-6R: soluble interleukin-6 receptor
sgp130: soluble glycoprotein 130
TBI: traumatic brain injury
VEH: vehicle
VP: visible platform
